# Crystal structure of 4-chloro-*N*-{5-(4-meth­oxyphen­yl)-4-[(4-meth­oxy­phen­yl)amino]-6-sulfanyl­idene-1,2,5,6-tetra­hydro-1,3,5-triazin-2-yl­idene}benzene­sulfonamide dimethyl sulfoxide disolvate

**DOI:** 10.1107/S2056989025010400

**Published:** 2025-11-25

**Authors:** Reham A. Mohamed-Ezzat, Galal H. Elgemeie, Peter G. Jones

**Affiliations:** a Chemistry of Natural and Microbial Products Department, Pharmaceutical and Drug Industries Research Institute, National Research Centre, Cairo, Egypt; b Chemistry Department, Faculty of Science, Helwan University, Cairo, Egypt; c Institut für Anorganische und Analytische Chemie, Technische Universität Braunschweig, Hagenring 30, D-38106 Braunschweig, Germany; Vienna University of Technology, Austria

**Keywords:** crystal structure, triazine, DMSO, hydrogen bonds, H⋯π contacts, O⋯π contacts

## Abstract

In the structure of the title compound, C_23_H_20_ClN_5_O_4_S_2_·2C_2_H_6_OS, the two DMSO solvent mol­ecules are hydrogen bonded to the two NH groups of the main mol­ecule. The (modified) triazine ring is planar and is approximately perpendicular to its directly bonded meth­oxy­phenyl substituent. The bond lengths and angles indicate extensive delocalization of the multiple bonding. The packing is characterized by a large number of borderline contacts, especially ‘weak’ hydrogen bonds. There is no π–π stacking. The centroid of the triazine ring is involved in two short contacts, namely 2.63 Å to a phenyl hydrogen atom and 3.0509 (9) Å to a meth­oxy oxygen atom.

## Abstract 2

The (modified) triazine ring in the title compound is planar. The packing involves classical and ‘weak’ hydrogen bonds, together with π contacts to the triazine ring.

## 1. Chemical context

Triazine sulfonamides represent a recently developed class of heterocyclic compounds known for their diverse biological activities (Kciuk *et al.*, 2023 ▸). These compounds combine the pharmacological benefits of both triazine and sulfonamide moieties, resulting in an enhanced therapeutic potential.

Sulfonamides represent a crucial class of drugs with diverse pharmacological activities. They exhibit carbonic anhydrase inhibition (Supuran *et al.*, 2008 ▸) and possess anti­bacterial, anti­malarial, high-ceiling diuretic, anti­hypertensive, hypoglycemic, anti-inflammatory, anti­protozoal, anti­thyroid, anti­glaucoma and anti­tumour properties (Mojzych *et al.*, 2015 ▸). Hetero-aromatic sulfonamides have been found to counteract tumour acidification, thereby inhibiting cancer cell growth and preventing tumour invasion facilitated by carbonic anhydrases (Wykoff *et al.*, 2000 ▸). Meanwhile, triazines are a significant class of heterocyclic compounds in medicinal chemistry, recognized for their potent anti­cancer and anti­bacterial properties (Smirnov *et al.*, 1997 ▸). Additionally, they have been explored for their anti-SARS-CoV-2 effects, part of a search for new therapeutic agents (Mohamed-Ezzat & Elgemeie, 2024*a* ▸,*b* ▸). Their expanding role underscores their potential as a new generation of therapeutic agents for various diseases.

**Figure 1:**
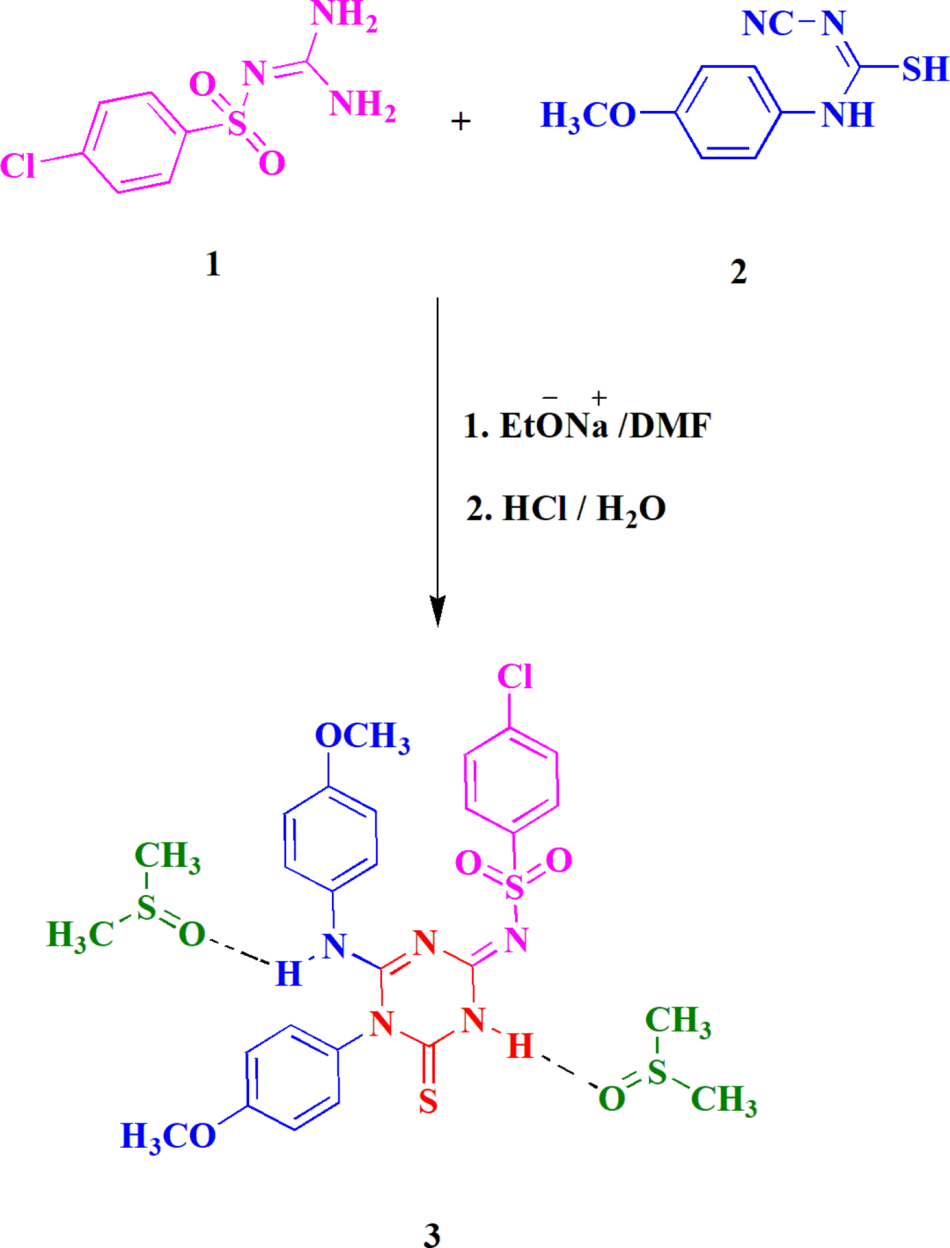
The synthesis of the title compound **3**.

Herein, we have synthesized and characterized a new triazine­thione sulfonamide, the title compound **3**, using a novel synthetic approach (Fig. 1 ▸) that utilizes dimethyl cyano­carboimidodi­thio­ate as a key precursor of **2**. This highly reactive compound has demonstrated remarkable effectiveness in the synthesis of various heterocycles (Elgemeie & Mohamed, 2014*a* ▸,*b* ▸; Mohamed-Ezzat & Elgemeie, 2023 ▸), particularly pyrimidine derivatives (Mohamed-Ezzat *et al.*, 2024 ▸). Its unique reactivity provides an efficient pathway for generating structurally varied triazine sulfonamides, expanding the chemical space for potential pharmaceutical applications. The structure of **3** was first derived via spectroscopic techniques; the ^1^H NMR spectrum revealed the presence of a singlet at 3.78 ppm, which indicated the presence of two sets of meth­oxy protons. The singlet at 8.82 ppm probably corresponds to an NH proton, and a significantly downfield singlet at 13.03 ppm is characteristic of an NH proton involved in strong hydrogen bonding. As part of our ongoing research to explore the structural and functional aspects of these compounds, we have undertaken a single-crystal X-ray diffraction study to confirm the chemical nature of **3** and to determine its precise mol­ecular conformation. Compound **3** crystallized as its di­methyl­sulfoxide disolvate.
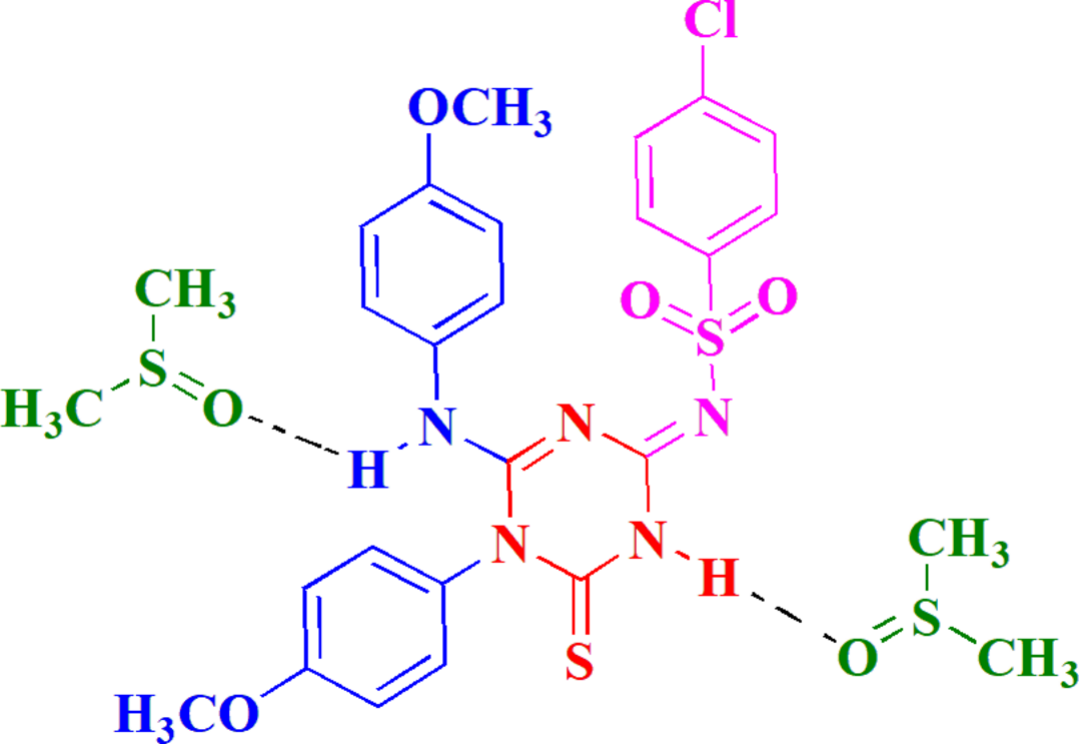


## 2. Structural commentary

**Figure 2:**
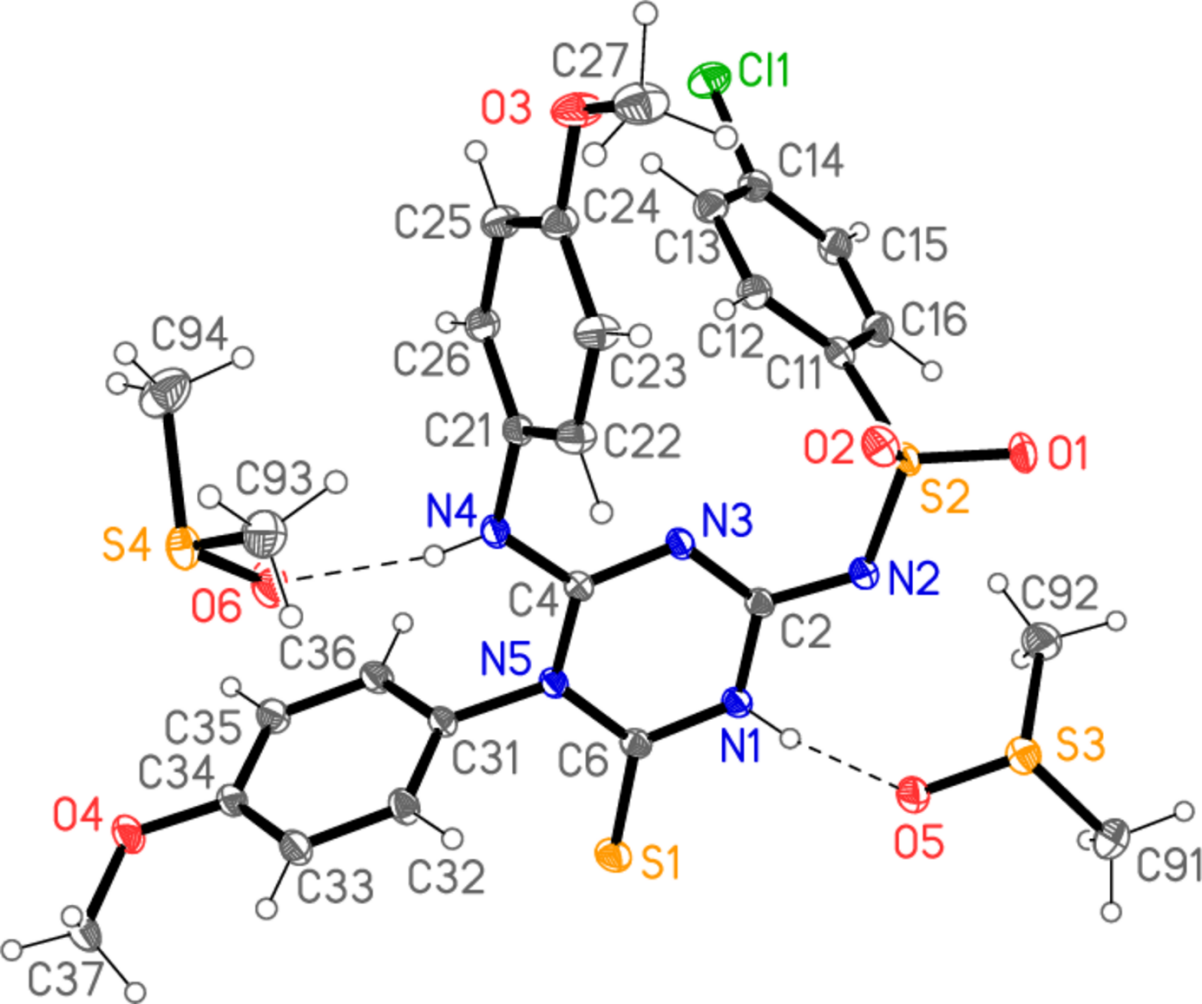
The structure of compound **3** in the crystal, including the two solvent mol­ecules. Ellipsoids are drawn at the 50% probability level. Dashed lines indicate classical hydrogen bonds.

The structure of compound **3** is shown in Fig. 2 ▸, including the two DMSO solvent mol­ecules, which are hydrogen bonded to the two NH groups at N1 and N4. Selected bond lengths and angles are given in Table 1 ▸. The C=S bond length of 1.6499 (10) Å is slightly shorter than the ‘standard’ value of 1.681 (20) Å given in the extensive database analysis of bond lengths by Allen *et al.* (1987 ▸) for thio­urea-type groups. The central triazine ring is essentially planar (r.m.s.d. 0.025 Å) and subtends an angle of 79.60 (3)° with the directly bonded meth­oxy­phenyl group at N5. Inspection of the triazine bond lengths and angles shows that the resonance form given in the Scheme must be an oversimplification, ignoring as it does the probable extensive delocalization of the multiple bonding; thus the C—N bond lengths vary over a quite narrow range [1.3234 (12)–1.3930 (13) Å], with the formally double bond N3=C4 being (just) the shortest. The ring angles vary over the range 114.62 (8)–123.36 (8)°; the narrowest by far is that at C6, which bears the thio­nic sulfur atom S1.

## 3. Supra­molecular features

**Figure 3:**
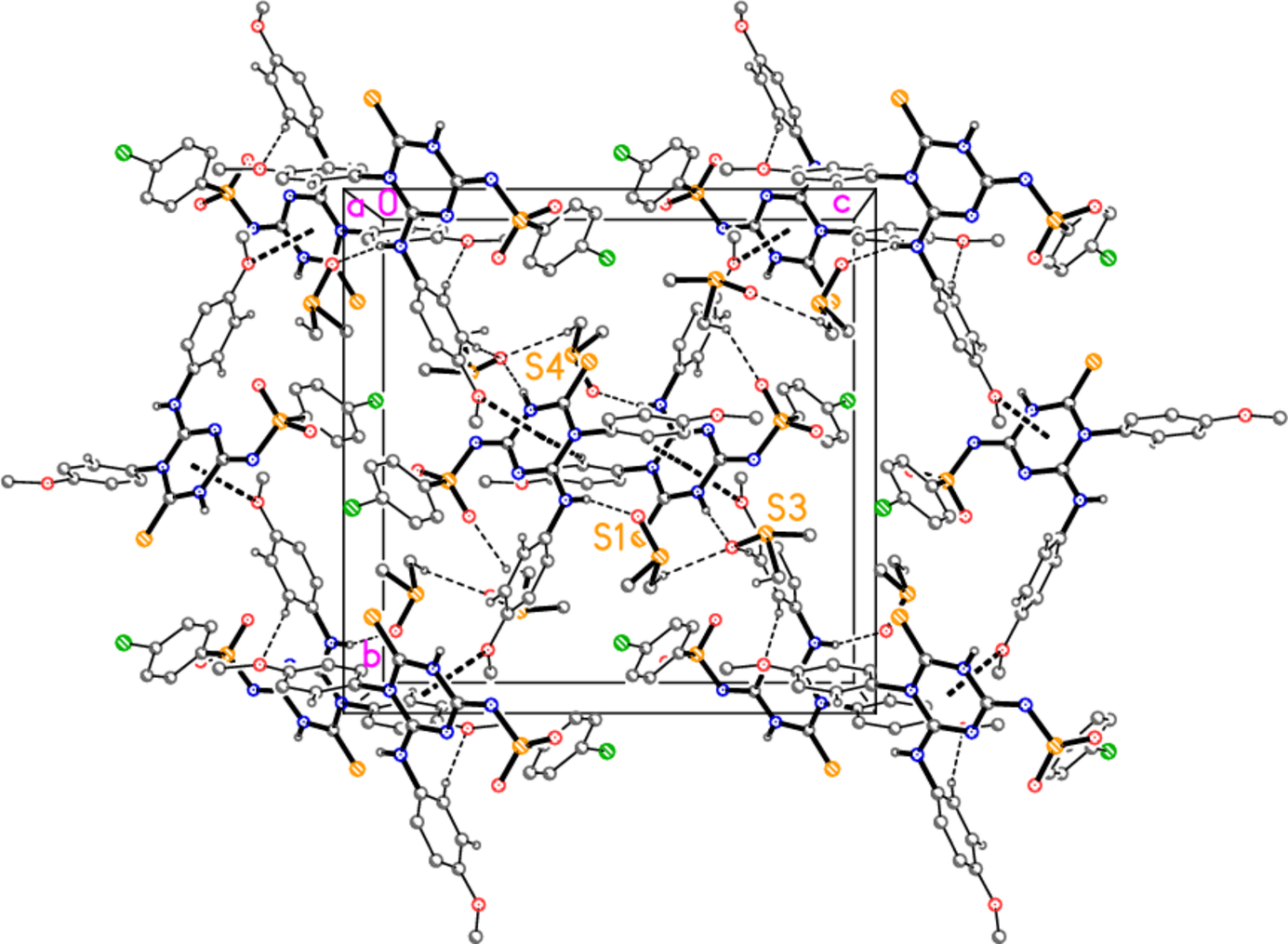
Packing of compound **3** viewed parallel to the *a* axis. Hydrogen bonds (both classical and ‘weak’) are shown as thin dashed lines. Contacts to the triazine ring centroids are shown as thick dashed lines. Sulfur atoms are labelled to identify the asymmetric unit.

Hydrogen bonds are listed in Table 2 ▸. Classical hydrogen bonds are observed from both NH functions to the solvent oxygen atoms (Fig. 2 ▸). The packing is also characterized by a large number of borderline contacts, such as H92*C*⋯N2 (2.62 Å) and N2⋯S3 [3.2470 (9) Å] within the asymmetric unit, and S1⋯Cl1(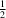 − *x*, 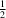 + *y*, 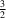 − *z*) [3.6356 (4) Å]. Furthermore, many of the ‘weak’ hydrogen bonds with C—H donors (Table 2 ▸) are borderline in terms both of length and angle. There are no π–π contacts that would indicate stacking; no inter­centroid distance is < 4 Å. Perhaps the most inter­esting of the contacts are those to the centroid (*Cg*) of the triazine ring, namely H32⋯*Cg*(1 − *x*, 1 − *y*, 1 − *z*) (2.63 Å), with an angle C32—H32⋯*Cg* of 151°, and O3⋯*Cg*(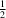 − *x*, −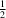 + *y*, 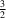 − *z*) [3.0509 (9) Å], with an angle C24—O3⋯*Cg* of 138°. All attempts to provide a reasonably comprehensive packing diagram lead to complex diagrams that are difficult to inter­pret, but Fig. 3 ▸ gives an impression of the packing viewed parallel to the *a* axis, with particular emphasis on the *Cg* inter­actions. The C—H⋯O contacts that are shown are those from H25, H26, H32, H91*A* and H93*A* (*cf.* Table 2 ▸).

## 4. Database survey

The searches (of version 6.00 of the Cambridge Database; Groom *et al.*, 2016 ▸) employed the routine ConQuest (version 2025.1.1; Bruno *et al.*, 2002 ▸). A search for non-annelated *sym*-triazine-type rings with a similar connectivity to that of **3** (one two-coordinate nitro­gen atom, all other atoms three-coordinate, no restrictions on bond order), but excluding 2,4,6-tri­thioxo substitution, gave only one hit, namely, 6-di­ethyl­amino-2,4-di­thioxo-1*H*,3*H*-1,3,5-triazine (refcode WIVHUE; Coxall *et al.*, 2000 ▸). This however has two exocyclic formal C=S double bonds and so is not closely related to **3**, suggesting that **3** is a novel type of substituted (and partially reduced) triazine.

The parent 2,4,6-tri­thioxo derivative, C_3_H_3_N_3_S_3_, also known as tri­thia­cyanuric acid, is known as two crystalline forms (CEHQEM, Guo *et al.*, 2006 ▸ and CEHQEM01, Brito *et al.*, 2010 ▸).

## 5. Synthesis and crystallization

A mixture of *p*-chloro­benzene­sulfon­ylguanidine (0.01 mol) **1** and 1-cyano-3-(4-meth­oxy­phen­yl)iso­thio­urea (0.01 mol) **2** was refluxed in dry dimethyl formamide (20 ml containing sodium ethoxide (0.01 mol) for 2 h. The reaction mixture was then poured into ice water and neutralized with hydro­chloric acid. The resulting precipitate was filtered off, thoroughly washed with water, dried, and recrystallized from dimethyl sulfoxide, yielding the dimethyl sulfoxide disolvate of compound **3** as yellow crystals in 71% yield., m.p. 554–555 K; ^1^H NMR (500 MHz, DMSO-*d_6_*): δ (ppm) 3.78 (*s*, 6H, OCH_3_), 6.95–6.97 (*d*, 2H, Ar—H), 7.02–7.03(*d*, 2H, Ar—H), 7.14–7.15 (*m*, 4H, Ar—H), 7.27–7.29(*d*, 2H, Ar—H), 7.36–7.37(*d*, 2H, Ar—H), 8.82 (*s*, 1H, NH), 13.03 (*s*, 1H, NH);^13^C NMR (500 MHz, DMSO-*d*_6_): δ (ppm) 178.75, 160.70, 158.36, 153.56, 153.29, 142.25, 136.23, 131.05, 129.65, 129.42, 128.79, 128.34, 115.91, 113.92, 100.00, 55.98, 55.86. Analysis: calc. for C_29_H_36_ClN_5_O_6_S_4_ (714.34): C 48.76, H 5.08, Cl 4.96, N 9.80, S 17.96. Found: C 48.70, H 5.01, Cl 4.94, N 9.80, S 17.91%.

## 6. Refinement

Details of data collection and structure refinement are summarized in Table 3 ▸. The atom numbering of the central triazine ring is the IUPAC numbering; peripheral rings are denoted as C*n*1–C*n*6, with *n* = 1–3. The hydrogen atoms of the NH groups were refined freely. Methyl H atoms were refined as parts of idealized rigid groups with C—H = 0.98 Å, H—C—H = 109.5°, allowed to rotate but not tip (‘AFIX 137’). Other hydrogen atoms were included using a riding model starting from calculated positions (C—H_arom_ = 0.95 Å). The *U*(H) values were fixed at 1.5 × *U*_eq_ of the parent carbon atoms for methyl H atoms and 1.2 × *U*_eq_ for the other H atoms.

## Supplementary Material

Crystal structure: contains datablock(s) I, global. DOI: 10.1107/S2056989025010400/wm5778sup1.cif

Structure factors: contains datablock(s) I. DOI: 10.1107/S2056989025010400/wm5778Isup2.hkl

Supporting information file. DOI: 10.1107/S2056989025010400/wm5778Isup3.cml

CCDC reference: 2504050

Additional supporting information:  crystallographic information; 3D view; checkCIF report

## Figures and Tables

**Table 1:** Selected geometric parameters (Å, °)

N1—C6	1.3552 (12)	C4—N5	1.3870 (12)
N1—C2	1.3761 (12)	N5—C6	1.3930 (13)
C2—N2	1.3226 (12)	N5—C31	1.4429 (12)
C2—N3	1.3382 (12)	C6—S1	1.6499 (10)
N3—C4	1.3234 (12)	N2—S2	1.6121 (9)
C4—N4	1.3327 (12)		
			
C6—N1—C2	123.36 (8)	C4—N5—C6	120.38 (8)
N2—C2—N3	125.07 (9)	N1—C6—N5	114.62 (8)
C4—N3—C2	117.72 (8)	N1—C6—S1	121.94 (7)
N3—C4—N5	122.66 (8)	N5—C6—S1	123.44 (7)

**Table 2:** Hydrogen-bond geometry (Å, °)

*D*—H⋯*A*	*D*—H	H⋯*A*	*D*⋯*A*	*D*—H⋯*A*
N1—H01⋯O5	0.87 (2)	1.89 (2)	2.7510 (12)	171.3 (19)
N4—H04⋯O6	0.78 (2)	2.00 (2)	2.7539 (12)	161 (2)
C25—H25⋯O5^i^	0.95	2.64	3.5542 (14)	161
C26—H26⋯O4^ii^	0.95	2.54	3.4726 (14)	166
C33—H33⋯N2^iii^	0.95	2.68	3.5630 (13)	155
C91—H91*A*⋯O2^iv^	0.98	2.43	3.2398 (16)	140
C92—H92*C*⋯N2	0.98	2.62	3.3263 (16)	129
C93—H93*A*⋯O5^iii^	0.98	2.54	3.3080 (16)	135

Symmetry codes: (i) 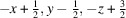; (ii) 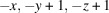; (iii) 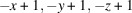; (iv) 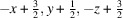.

**Table 3:** Experimental details

Crystal data	
Chemical formula	C_23_H_20_ClN_5_O_4_S_2_·2C_2_H_6_OS
*M*_r_	686.26
Crystal system, space group	Monoclinic, *P*2_1_/*n*
Temperature (K)	100
*a*, *b*, *c* (Å)	11.1601 (2), 16.7646 (3), 17.0386 (3)
β (°)	91.5422 (16)
*V* (Å^3^)	3186.67 (10)
*Z*	4
Radiation type	Mo *K*α
μ (mm^−1^)	0.43
Crystal size (mm)	0.2 × 0.1 × 0.1

Data collection	
Diffractometer	XtaLAB Synergy
Absorption correction	Multi-scan (*CrysAlis PRO*; Rigaku OD, 2024 ▸)
*T*_min_, *T*_max_	0.626, 1.000
No. of measured, independent and observed [*I* > 2σ(*I*)] reflections	304849, 15458, 12448
*R*_int_	0.078
θ values (°)	θ_max_ = 36.3, θ_min_ = 2.2
(sin θ/λ)_max_ (Å^−1^)	0.833

Refinement	
*R*[*F*^2^ > 2σ(*F*^2^)], *wR*(*F*^2^), *S*	0.039, 0.118, 1.04
No. of reflections	15458
No. of parameters	402
H-atom treatment	H atoms treated by a mixture of independent and constrained refinement
Δρ_max_, Δρ_min_ (e Å^−3^)	0.99, −0.65

Computer programs: *CrysAlis PRO* (Rigaku OD, 2024 ▸), *SHELXT* (Sheldrick, 2015*a* ▸), *SHELXL2019/3* (Sheldrick, 2015*b* ▸), *XP* (Bruker, 1998 ▸) and *publCIF* (Westrip, 2010 ▸).

## supplementary crystallographic information

### 4-Chloro-N-{5-(4-methoxyphenyl)-4-[(4-methoxyphenyl)amino]-6-sulfanylidene-1,2,5,6-tetrahydro-1,3,5-triazin-2-ylidene}benzenesulfonamide dimethyl sulfoxide disolvate Crystal data

**Table:**

C_23_H_20_ClN_5_O_4_S_2_·2C_2_H_6_OS	*F*(000) = 1432
*M**_r_* = 686.26	*D*_x_ = 1.430 Mg m^−^^3^
Monoclinic, *P*2_1_/*n*	Mo *K*α radiation, λ = 0.71073 Å
*a* = 11.1601 (2) Å	Cell parameters from 121720 reflections
*b* = 16.7646 (3) Å	θ = 2.1–38.3°
*c* = 17.0386 (3) Å	µ = 0.43 mm^−^^1^
β = 91.5422 (16)°	*T* = 100 K
*V* = 3186.67 (10) Å^3^	Block, pale yellow
*Z* = 4	0.2 × 0.1 × 0.1 mm

### 4-Chloro-N-{5-(4-methoxyphenyl)-4-[(4-methoxyphenyl)amino]-6-sulfanylidene-1,2,5,6-tetrahydro-1,3,5-triazin-2-ylidene}benzenesulfonamide dimethyl sulfoxide disolvate Data collection

**Table:**

XtaLAB Synergy diffractometer	15458 independent reflections
Radiation source: micro-focus sealed X-ray tube, PhotonJet (Mo) X-ray Source	12448 reflections with *I* > 2σ(*I*)
Mirror monochromator	*R*_int_ = 0.078
Detector resolution: 10.0000 pixels mm^-1^	θ_max_ = 36.3°, θ_min_ = 2.2°
ω scans	*h* = −18→18
Absorption correction: multi-scan (CrysAlisPro; Rigaku OD, 2024)	*k* = −27→27
*T*_min_ = 0.626, *T*_max_ = 1.000	*l* = −28→28
304849 measured reflections	

### 4-Chloro-N-{5-(4-methoxyphenyl)-4-[(4-methoxyphenyl)amino]-6-sulfanylidene-1,2,5,6-tetrahydro-1,3,5-triazin-2-ylidene}benzenesulfonamide dimethyl sulfoxide disolvate Refinement

**Table:**

Refinement on *F*^2^	Primary atom site location: dual
Least-squares matrix: full	Hydrogen site location: mixed
*R*[*F*^2^ > 2σ(*F*^2^)] = 0.039	H atoms treated by a mixture of independent and constrained refinement
*wR*(*F*^2^) = 0.118	*w* = 1/[σ^2^(*F*_o_^2^) + (0.0629*P*)^2^ + 1.4684*P*] where *P* = (*F*_o_^2^ + 2*F*_c_^2^)/3
*S* = 1.04	(Δ/σ)_max_ = 0.002
15458 reflections	Δρ_max_ = 0.99 e Å^−^^3^
402 parameters	Δρ_min_ = −0.65 e Å^−^^3^
0 restraints	

### 4-Chloro-N-{5-(4-methoxyphenyl)-4-[(4-methoxyphenyl)amino]-6-sulfanylidene-1,2,5,6-tetrahydro-1,3,5-triazin-2-ylidene}benzenesulfonamide dimethyl sulfoxide disolvate Fractional atomic coordinates and isotropic or equivalent isotropic displacement parameters (Å^2^)

**Table:**

	*x*	*y*	*z*	*U*_iso_*/*U*_eq_	
N1	0.47353 (8)	0.58343 (5)	0.66665 (5)	0.01437 (13)	
H01	0.5239 (18)	0.6196 (12)	0.6830 (12)	0.029 (5)*	
C2	0.46029 (8)	0.51624 (6)	0.71200 (5)	0.01295 (14)	
N3	0.38980 (7)	0.45613 (5)	0.68754 (5)	0.01356 (13)	
C4	0.33299 (8)	0.46390 (5)	0.61878 (5)	0.01240 (14)	
N5	0.33665 (7)	0.53350 (5)	0.57490 (5)	0.01313 (13)	
C6	0.41030 (9)	0.59652 (6)	0.59885 (5)	0.01407 (14)	
S1	0.42309 (3)	0.68012 (2)	0.54867 (2)	0.02055 (6)	
N2	0.52430 (8)	0.51712 (5)	0.77846 (5)	0.01537 (14)	
S2	0.52474 (2)	0.43949 (2)	0.83430 (2)	0.01461 (5)	
O1	0.60234 (7)	0.46032 (5)	0.90037 (5)	0.02037 (14)	
O2	0.55298 (7)	0.36661 (5)	0.79378 (5)	0.01899 (14)	
C11	0.37858 (9)	0.43008 (6)	0.87138 (5)	0.01500 (15)	
C12	0.30220 (10)	0.37045 (6)	0.84289 (6)	0.01762 (16)	
H12	0.325617	0.337355	0.800720	0.021*	
C13	0.19120 (10)	0.35977 (6)	0.87678 (6)	0.01858 (17)	
H13	0.137630	0.319727	0.857770	0.022*	
C14	0.16000 (9)	0.40871 (6)	0.93896 (6)	0.01760 (16)	
Cl1	0.02569 (3)	0.39127 (2)	0.98569 (2)	0.02459 (6)	
C15	0.23448 (10)	0.46954 (7)	0.96660 (6)	0.01943 (17)	
H15	0.210379	0.503192	1.008162	0.023*	
C16	0.34516 (9)	0.48021 (7)	0.93217 (6)	0.01779 (16)	
H16	0.397519	0.521437	0.950060	0.021*	
N4	0.26820 (8)	0.40306 (5)	0.59052 (5)	0.01455 (13)	
H04	0.2355 (18)	0.4054 (12)	0.5496 (12)	0.029 (5)*	
C21	0.26962 (9)	0.32737 (6)	0.63021 (6)	0.01367 (14)	
C22	0.37147 (9)	0.28023 (6)	0.62974 (6)	0.01817 (17)	
H22	0.440980	0.298772	0.604424	0.022*	
C23	0.37275 (10)	0.20573 (6)	0.66615 (7)	0.01967 (18)	
H23	0.442689	0.173467	0.665353	0.024*	
C24	0.27094 (9)	0.17880 (6)	0.70373 (6)	0.01678 (16)	
C25	0.16818 (9)	0.22643 (6)	0.70397 (7)	0.01812 (17)	
H25	0.098383	0.208033	0.729058	0.022*	
C26	0.16782 (9)	0.30061 (6)	0.66759 (6)	0.01662 (16)	
H26	0.098076	0.333065	0.668247	0.020*	
O3	0.26355 (8)	0.10742 (5)	0.74176 (6)	0.02301 (16)	
C27	0.36639 (11)	0.05705 (7)	0.74156 (9)	0.0266 (2)	
H27A	0.387903	0.045874	0.687268	0.040*	
H27B	0.348497	0.006874	0.768326	0.040*	
H27C	0.433499	0.083764	0.768946	0.040*	
C31	0.26734 (8)	0.54095 (6)	0.50255 (5)	0.01336 (14)	
C32	0.32259 (9)	0.52846 (7)	0.43192 (6)	0.01704 (16)	
H32	0.404897	0.514041	0.431584	0.020*	
C33	0.25820 (9)	0.53690 (7)	0.36132 (6)	0.01810 (17)	
H33	0.295898	0.528346	0.312703	0.022*	
C34	0.13775 (9)	0.55805 (6)	0.36307 (6)	0.01629 (16)	
C35	0.08205 (9)	0.56978 (7)	0.43453 (6)	0.01846 (17)	
H35	−0.000490	0.583544	0.435161	0.022*	
C36	0.14690 (9)	0.56139 (6)	0.50460 (6)	0.01686 (16)	
H36	0.109425	0.569525	0.553360	0.020*	
O4	0.06674 (8)	0.56915 (6)	0.29744 (5)	0.02212 (15)	
C37	0.12332 (12)	0.56460 (9)	0.22322 (7)	0.0279 (2)	
H37A	0.065073	0.578052	0.181219	0.042*	
H37B	0.153254	0.510340	0.215169	0.042*	
H37C	0.190387	0.602301	0.222418	0.042*	
S3	0.71264 (3)	0.66372 (2)	0.79923 (2)	0.01976 (5)	
O5	0.62909 (8)	0.69246 (5)	0.73409 (5)	0.02336 (16)	
C91	0.80784 (12)	0.74656 (9)	0.82165 (8)	0.0293 (2)	
H91A	0.861018	0.756117	0.777786	0.044*	
H91B	0.856015	0.734981	0.869232	0.044*	
H91C	0.758806	0.794095	0.830267	0.044*	
C92	0.62878 (13)	0.66821 (8)	0.88676 (7)	0.0272 (2)	
H92A	0.589148	0.720221	0.890062	0.041*	
H92B	0.682895	0.661016	0.932465	0.041*	
H92C	0.568228	0.625863	0.885850	0.041*	
S4	0.13116 (3)	0.29499 (2)	0.40610 (2)	0.02286 (6)	
O6	0.15339 (10)	0.37283 (5)	0.44855 (6)	0.02729 (18)	
C93	0.25565 (12)	0.23122 (8)	0.42623 (8)	0.0270 (2)	
H93A	0.326790	0.252644	0.401137	0.041*	
H93B	0.238214	0.177803	0.405552	0.041*	
H93C	0.270522	0.228098	0.483098	0.041*	
C94	0.02764 (14)	0.24312 (9)	0.46618 (9)	0.0336 (3)	
H94A	0.053700	0.247495	0.521399	0.050*	
H94B	0.024812	0.186776	0.450927	0.050*	
H94C	−0.052281	0.266690	0.459117	0.050*	

### 4-Chloro-N-{5-(4-methoxyphenyl)-4-[(4-methoxyphenyl)amino]-6-sulfanylidene-1,2,5,6-tetrahydro-1,3,5-triazin-2-ylidene}benzenesulfonamide dimethyl sulfoxide disolvate Atomic displacement parameters (Å^2^)

**Table:**

	*U*^11^	*U*^22^	*U*^33^	*U*^12^	*U*^13^	*U*^23^
N1	0.0170 (3)	0.0140 (3)	0.0120 (3)	−0.0016 (3)	−0.0005 (3)	0.0003 (2)
C2	0.0137 (3)	0.0141 (3)	0.0110 (3)	0.0001 (3)	0.0006 (3)	−0.0004 (3)
N3	0.0157 (3)	0.0140 (3)	0.0108 (3)	−0.0006 (2)	−0.0017 (2)	0.0006 (2)
C4	0.0133 (3)	0.0126 (3)	0.0112 (3)	0.0006 (3)	0.0001 (3)	0.0002 (3)
N5	0.0151 (3)	0.0127 (3)	0.0115 (3)	0.0001 (2)	−0.0014 (2)	0.0014 (2)
C6	0.0160 (4)	0.0137 (3)	0.0126 (3)	0.0002 (3)	0.0010 (3)	−0.0002 (3)
S1	0.02776 (13)	0.01500 (10)	0.01873 (11)	−0.00342 (9)	−0.00241 (9)	0.00438 (8)
N2	0.0168 (3)	0.0179 (3)	0.0114 (3)	−0.0024 (3)	−0.0015 (3)	0.0005 (3)
S2	0.01483 (9)	0.01820 (10)	0.01069 (9)	0.00036 (7)	−0.00178 (7)	0.00067 (7)
O1	0.0184 (3)	0.0290 (4)	0.0134 (3)	−0.0012 (3)	−0.0053 (2)	0.0004 (3)
O2	0.0207 (3)	0.0194 (3)	0.0169 (3)	0.0040 (3)	0.0001 (3)	−0.0004 (3)
C11	0.0166 (4)	0.0170 (4)	0.0113 (3)	−0.0002 (3)	−0.0007 (3)	0.0009 (3)
C12	0.0203 (4)	0.0180 (4)	0.0145 (4)	−0.0022 (3)	0.0007 (3)	−0.0016 (3)
C13	0.0198 (4)	0.0182 (4)	0.0178 (4)	−0.0032 (3)	0.0013 (3)	−0.0010 (3)
C14	0.0171 (4)	0.0198 (4)	0.0160 (4)	−0.0001 (3)	0.0014 (3)	0.0013 (3)
Cl1	0.02102 (11)	0.02603 (13)	0.02710 (13)	−0.00235 (9)	0.00780 (9)	−0.00101 (10)
C15	0.0194 (4)	0.0224 (4)	0.0165 (4)	−0.0001 (3)	0.0010 (3)	−0.0035 (3)
C16	0.0177 (4)	0.0208 (4)	0.0149 (4)	−0.0015 (3)	−0.0001 (3)	−0.0027 (3)
N4	0.0175 (3)	0.0133 (3)	0.0126 (3)	−0.0014 (3)	−0.0036 (3)	0.0010 (2)
C21	0.0153 (4)	0.0128 (3)	0.0128 (3)	−0.0009 (3)	−0.0016 (3)	0.0003 (3)
C22	0.0167 (4)	0.0166 (4)	0.0214 (4)	0.0012 (3)	0.0032 (3)	0.0040 (3)
C23	0.0170 (4)	0.0170 (4)	0.0253 (5)	0.0029 (3)	0.0048 (3)	0.0046 (3)
C24	0.0169 (4)	0.0137 (4)	0.0198 (4)	−0.0003 (3)	0.0008 (3)	0.0022 (3)
C25	0.0149 (4)	0.0172 (4)	0.0223 (4)	−0.0002 (3)	0.0020 (3)	0.0039 (3)
C26	0.0143 (4)	0.0164 (4)	0.0191 (4)	0.0005 (3)	−0.0003 (3)	0.0026 (3)
O3	0.0204 (3)	0.0161 (3)	0.0329 (4)	0.0015 (3)	0.0054 (3)	0.0085 (3)
C27	0.0236 (5)	0.0182 (4)	0.0382 (6)	0.0049 (4)	0.0055 (4)	0.0084 (4)
C31	0.0139 (3)	0.0151 (3)	0.0110 (3)	0.0016 (3)	−0.0006 (3)	0.0013 (3)
C32	0.0136 (4)	0.0246 (4)	0.0129 (4)	0.0036 (3)	−0.0002 (3)	−0.0002 (3)
C33	0.0156 (4)	0.0262 (5)	0.0126 (4)	0.0024 (3)	0.0003 (3)	0.0011 (3)
C34	0.0147 (4)	0.0198 (4)	0.0143 (4)	0.0004 (3)	−0.0018 (3)	0.0036 (3)
C35	0.0133 (4)	0.0247 (5)	0.0174 (4)	0.0034 (3)	−0.0002 (3)	0.0035 (3)
C36	0.0144 (4)	0.0218 (4)	0.0146 (4)	0.0033 (3)	0.0020 (3)	0.0024 (3)
O4	0.0185 (3)	0.0320 (4)	0.0156 (3)	0.0016 (3)	−0.0045 (3)	0.0049 (3)
C37	0.0270 (5)	0.0425 (7)	0.0140 (4)	−0.0014 (5)	−0.0042 (4)	0.0038 (4)
S3	0.02222 (12)	0.01785 (11)	0.01912 (11)	0.00041 (8)	−0.00108 (9)	−0.00122 (8)
O5	0.0262 (4)	0.0240 (4)	0.0195 (3)	−0.0074 (3)	−0.0057 (3)	0.0031 (3)
C91	0.0256 (5)	0.0328 (6)	0.0291 (6)	−0.0112 (5)	−0.0048 (4)	0.0011 (5)
C92	0.0314 (6)	0.0310 (6)	0.0191 (5)	−0.0068 (5)	0.0010 (4)	−0.0008 (4)
S4	0.02710 (13)	0.02552 (13)	0.01577 (11)	−0.00446 (10)	−0.00265 (9)	−0.00470 (9)
O6	0.0388 (5)	0.0202 (4)	0.0223 (4)	−0.0056 (3)	−0.0095 (3)	−0.0019 (3)
C93	0.0294 (6)	0.0268 (5)	0.0248 (5)	−0.0034 (4)	0.0002 (4)	−0.0027 (4)
C94	0.0326 (6)	0.0335 (6)	0.0352 (7)	−0.0109 (5)	0.0106 (5)	−0.0137 (5)

### 4-Chloro-N-{5-(4-methoxyphenyl)-4-[(4-methoxyphenyl)amino]-6-sulfanylidene-1,2,5,6-tetrahydro-1,3,5-triazin-2-ylidene}benzenesulfonamide dimethyl sulfoxide disolvate Geometric parameters (Å, º)

**Table:**

N1—C6	1.3552 (12)	C26—H26	0.9500
N1—C2	1.3761 (12)	O3—C27	1.4250 (14)
N1—H01	0.87 (2)	C27—H27A	0.9800
C2—N2	1.3226 (12)	C27—H27B	0.9800
C2—N3	1.3382 (12)	C27—H27C	0.9800
N3—C4	1.3234 (12)	C31—C32	1.3829 (13)
C4—N4	1.3327 (12)	C31—C36	1.3885 (13)
C4—N5	1.3870 (12)	C32—C33	1.3916 (14)
N5—C6	1.3930 (13)	C32—H32	0.9500
N5—C31	1.4429 (12)	C33—C34	1.3914 (14)
C6—S1	1.6499 (10)	C33—H33	0.9500
N2—S2	1.6121 (9)	C34—O4	1.3657 (12)
S2—O2	1.4424 (8)	C34—C35	1.3958 (15)
S2—O1	1.4447 (8)	C35—C36	1.3864 (14)
S2—C11	1.7720 (10)	C35—H35	0.9500
C11—C16	1.3925 (14)	C36—H36	0.9500
C11—C12	1.3927 (14)	O4—C37	1.4308 (15)
C12—C13	1.3923 (15)	C37—H37A	0.9800
C12—H12	0.9500	C37—H37B	0.9800
C13—C14	1.3916 (15)	C37—H37C	0.9800
C13—H13	0.9500	S3—O5	1.5092 (9)
C14—C15	1.3898 (15)	S3—C92	1.7835 (13)
C14—Cl1	1.7407 (11)	S3—C91	1.7837 (13)
C15—C16	1.3931 (15)	C91—H91A	0.9800
C15—H15	0.9500	C91—H91B	0.9800
C16—H16	0.9500	C91—H91C	0.9800
N4—C21	1.4377 (12)	C92—H92A	0.9800
N4—H04	0.78 (2)	C92—H92B	0.9800
C21—C22	1.3846 (14)	C92—H92C	0.9800
C21—C26	1.3917 (14)	S4—O6	1.5093 (9)
C22—C23	1.3944 (14)	S4—C93	1.7790 (14)
C22—H22	0.9500	S4—C94	1.7895 (15)
C23—C24	1.3943 (15)	C93—H93A	0.9800
C23—H23	0.9500	C93—H93B	0.9800
C24—O3	1.3644 (13)	C93—H93C	0.9800
C24—C25	1.3975 (14)	C94—H94A	0.9800
C25—C26	1.3895 (14)	C94—H94B	0.9800
C25—H25	0.9500	C94—H94C	0.9800
			
C6—N1—C2	123.36 (8)	C24—O3—C27	117.37 (9)
C6—N1—H01	118.6 (13)	O3—C27—H27A	109.5
C2—N1—H01	118.1 (13)	O3—C27—H27B	109.5
N2—C2—N3	125.07 (9)	H27A—C27—H27B	109.5
N2—C2—N1	114.08 (8)	O3—C27—H27C	109.5
N3—C2—N1	120.84 (8)	H27A—C27—H27C	109.5
C4—N3—C2	117.72 (8)	H27B—C27—H27C	109.5
N3—C4—N4	119.13 (8)	C32—C31—C36	120.92 (9)
N3—C4—N5	122.66 (8)	C32—C31—N5	119.28 (8)
N4—C4—N5	118.20 (8)	C36—C31—N5	119.80 (8)
C4—N5—C6	120.38 (8)	C31—C32—C33	120.33 (9)
C4—N5—C31	120.70 (8)	C31—C32—H32	119.8
C6—N5—C31	118.89 (8)	C33—C32—H32	119.8
N1—C6—N5	114.62 (8)	C34—C33—C32	118.95 (9)
N1—C6—S1	121.94 (7)	C34—C33—H33	120.5
N5—C6—S1	123.44 (7)	C32—C33—H33	120.5
C2—N2—S2	119.29 (7)	O4—C34—C33	123.84 (9)
O2—S2—O1	116.33 (5)	O4—C34—C35	115.65 (9)
O2—S2—N2	113.50 (5)	C33—C34—C35	120.51 (9)
O1—S2—N2	104.89 (5)	C36—C35—C34	120.16 (9)
O2—S2—C11	108.08 (5)	C36—C35—H35	119.9
O1—S2—C11	106.37 (5)	C34—C35—H35	119.9
N2—S2—C11	107.10 (5)	C35—C36—C31	119.12 (9)
C16—C11—C12	121.25 (9)	C35—C36—H36	120.4
C16—C11—S2	118.63 (8)	C31—C36—H36	120.4
C12—C11—S2	120.02 (8)	C34—O4—C37	117.15 (9)
C13—C12—C11	119.40 (10)	O4—C37—H37A	109.5
C13—C12—H12	120.3	O4—C37—H37B	109.5
C11—C12—H12	120.3	H37A—C37—H37B	109.5
C14—C13—C12	118.92 (10)	O4—C37—H37C	109.5
C14—C13—H13	120.5	H37A—C37—H37C	109.5
C12—C13—H13	120.5	H37B—C37—H37C	109.5
C15—C14—C13	122.07 (10)	O5—S3—C92	105.89 (6)
C15—C14—Cl1	118.87 (8)	O5—S3—C91	105.17 (6)
C13—C14—Cl1	119.03 (8)	C92—S3—C91	96.37 (7)
C14—C15—C16	118.72 (10)	S3—C91—H91A	109.5
C14—C15—H15	120.6	S3—C91—H91B	109.5
C16—C15—H15	120.6	H91A—C91—H91B	109.5
C11—C16—C15	119.60 (10)	S3—C91—H91C	109.5
C11—C16—H16	120.2	H91A—C91—H91C	109.5
C15—C16—H16	120.2	H91B—C91—H91C	109.5
C4—N4—C21	120.43 (8)	S3—C92—H92A	109.5
C4—N4—H04	121.4 (15)	S3—C92—H92B	109.5
C21—N4—H04	117.6 (15)	H92A—C92—H92B	109.5
C22—C21—C26	119.94 (9)	S3—C92—H92C	109.5
C22—C21—N4	119.98 (9)	H92A—C92—H92C	109.5
C26—C21—N4	120.07 (9)	H92B—C92—H92C	109.5
C21—C22—C23	120.48 (9)	O6—S4—C93	108.05 (6)
C21—C22—H22	119.8	O6—S4—C94	104.26 (6)
C23—C22—H22	119.8	C93—S4—C94	96.35 (7)
C24—C23—C22	119.71 (9)	S4—C93—H93A	109.5
C24—C23—H23	120.1	S4—C93—H93B	109.5
C22—C23—H23	120.1	H93A—C93—H93B	109.5
O3—C24—C23	124.23 (9)	S4—C93—H93C	109.5
O3—C24—C25	116.08 (9)	H93A—C93—H93C	109.5
C23—C24—C25	119.69 (9)	H93B—C93—H93C	109.5
C26—C25—C24	120.17 (9)	S4—C94—H94A	109.5
C26—C25—H25	119.9	S4—C94—H94B	109.5
C24—C25—H25	119.9	H94A—C94—H94B	109.5
C25—C26—C21	120.01 (9)	S4—C94—H94C	109.5
C25—C26—H26	120.0	H94A—C94—H94C	109.5
C21—C26—H26	120.0	H94B—C94—H94C	109.5
			
C6—N1—C2—N2	−175.76 (9)	S2—C11—C16—C15	−174.73 (8)
C6—N1—C2—N3	5.65 (14)	C14—C15—C16—C11	−0.07 (16)
N2—C2—N3—C4	−178.89 (9)	N3—C4—N4—C21	−6.20 (14)
N1—C2—N3—C4	−0.47 (13)	N5—C4—N4—C21	174.62 (8)
C2—N3—C4—N4	175.77 (9)	C4—N4—C21—C22	−70.31 (13)
C2—N3—C4—N5	−5.08 (14)	C4—N4—C21—C26	111.15 (11)
N3—C4—N5—C6	5.81 (14)	C26—C21—C22—C23	0.35 (16)
N4—C4—N5—C6	−175.04 (9)	N4—C21—C22—C23	−178.19 (10)
N3—C4—N5—C31	−176.16 (9)	C21—C22—C23—C24	−0.42 (17)
N4—C4—N5—C31	3.00 (13)	C22—C23—C24—O3	−179.21 (11)
C2—N1—C6—N5	−4.77 (13)	C22—C23—C24—C25	0.56 (17)
C2—N1—C6—S1	176.46 (8)	O3—C24—C25—C26	179.16 (10)
C4—N5—C6—N1	−0.72 (13)	C23—C24—C25—C26	−0.63 (16)
C31—N5—C6—N1	−178.79 (8)	C24—C25—C26—C21	0.56 (16)
C4—N5—C6—S1	178.02 (7)	C22—C21—C26—C25	−0.41 (15)
C31—N5—C6—S1	−0.05 (12)	N4—C21—C26—C25	178.12 (9)
N3—C2—N2—S2	2.91 (14)	C23—C24—O3—C27	−1.09 (17)
N1—C2—N2—S2	−175.61 (7)	C25—C24—O3—C27	179.13 (11)
C2—N2—S2—O2	51.83 (9)	C4—N5—C31—C32	−98.61 (11)
C2—N2—S2—O1	179.86 (8)	C6—N5—C31—C32	79.46 (12)
C2—N2—S2—C11	−67.37 (9)	C4—N5—C31—C36	82.13 (12)
O2—S2—C11—C16	160.16 (8)	C6—N5—C31—C36	−99.80 (11)
O1—S2—C11—C16	34.55 (10)	C36—C31—C32—C33	0.56 (16)
N2—S2—C11—C16	−77.21 (9)	N5—C31—C32—C33	−178.69 (10)
O2—S2—C11—C12	−16.16 (10)	C31—C32—C33—C34	0.01 (17)
O1—S2—C11—C12	−141.77 (8)	C32—C33—C34—O4	178.94 (10)
N2—S2—C11—C12	106.47 (9)	C32—C33—C34—C35	−0.69 (17)
C16—C11—C12—C13	−1.20 (16)	O4—C34—C35—C36	−178.84 (10)
S2—C11—C12—C13	175.03 (8)	C33—C34—C35—C36	0.81 (17)
C11—C12—C13—C14	−0.61 (16)	C34—C35—C36—C31	−0.25 (16)
C12—C13—C14—C15	2.12 (17)	C32—C31—C36—C35	−0.44 (16)
C12—C13—C14—Cl1	−175.79 (8)	N5—C31—C36—C35	178.81 (9)
C13—C14—C15—C16	−1.78 (17)	C33—C34—O4—C37	−5.05 (16)
Cl1—C14—C15—C16	176.14 (8)	C35—C34—O4—C37	174.59 (11)
C12—C11—C16—C15	1.54 (16)		

### 4-Chloro-N-{5-(4-methoxyphenyl)-4-[(4-methoxyphenyl)amino]-6-sulfanylidene-1,2,5,6-tetrahydro-1,3,5-triazin-2-ylidene}benzenesulfonamide dimethyl sulfoxide disolvate Hydrogen-bond geometry (Å, º)

**Table:**

*D*—H···*A*	*D*—H	H···*A*	*D*···*A*	*D*—H···*A*
N1—H01···O5	0.87 (2)	1.89 (2)	2.7510 (12)	171.3 (19)
N4—H04···O6	0.78 (2)	2.00 (2)	2.7539 (12)	161 (2)
C13—H13···S1^i^	0.95	2.92	3.5211 (11)	122
C15—H15···O1^ii^	0.95	2.64	3.1008 (13)	110
C16—H16···O1^ii^	0.95	2.57	3.0640 (13)	113
C25—H25···O5^i^	0.95	2.64	3.5542 (14)	161
C26—H26···O4^iii^	0.95	2.54	3.4726 (14)	166
C33—H33···N2^iv^	0.95	2.68	3.5630 (13)	155
C91—H91*A*···O2^v^	0.98	2.43	3.2398 (16)	140
C92—H92*C*···N2	0.98	2.62	3.3263 (16)	129
C93—H93*A*···O5^iv^	0.98	2.54	3.3080 (16)	135

Symmetry codes: (i) −*x*+1/2, *y*−1/2, −*z*+3/2; (ii) −*x*+1, −*y*+1, −*z*+2; (iii) −*x*, −*y*+1, −*z*+1; (iv) −*x*+1, −*y*+1, −*z*+1; (v) −*x*+3/2, *y*+1/2, −*z*+3/2.

## References

[bb1] Allen, F. H., Kennard, O., Watson, D. G., Brammer, L., Orpen, A. G. & Taylor, R. (1987). *J. Chem. Soc. Perkin Trans. 2* pp. S1–S19.

[bb2] Brito, I., Albanez, J. & Bolte, M. (2010). *Acta Cryst.* E**66**, o2382–o2383. 10.1107/S1600536810033234 PMC3007918 21588719

[bb3] Bruker (1998). *XP*. Bruker Analytical X-Ray Instruments, Madison, Wisconsin, USA.

[bb4] Bruno, I. J., Cole, J. C., Edgington, P. R., Kessler, M., Macrae, C. F., McCabe, P., Pearson, J. & Taylor, R. (2002). *Acta Cryst.* B**58**, 389–397. 10.1107/s0108768102003324 12037360

[bb5] Coxall, R. A., Harris, S. G., Henderson, D. K., Parsons, S., Tasker, P. A. & Winpenny, R. E. P. (2000). *J. Chem. Soc. Dalton Trans.* pp. 2349–2356.

[bb6] Elgemeie, G. H. & Mohamed, R. A. (2014*a*). *Heterocycl. Commun.* **20**, 257–269.

[bb7] Elgemeie, G. H. & Mohamed, R. A. (2014*b*). *Heterocycl. Commun.* **20**, 313–331.

[bb8] Groom, C. R., Bruno, I. J., Lightfoot, M. P. & Ward, S. C. (2016). *Acta Cryst.* B**72**, 171–179. 10.1107/S2052520616003954 PMC4822653 27048719

[bb9] Guo, F., Cheung, E. Y., Harris, K. D. M. & Pedireddi, V. R. (2006). *Cryst. Growth Des.* **6**, 846–848.

[bb10] Kciuk, M., Marciniak, B., Celik, I., Zerroug, E., Dubey, A., Sundaraj, R., Mujwar, S., Bukowski, K., Mojzych, M. & Kontek, R. (2023). *Int. J. Mol. Sci.* **24**, 10959 (https://doi. org/10.3390/ijms241310959). 10.3390/ijms241310959 PMC10342037 37446136

[bb11] Mohamed-Ezzat, R. A. & Elgemeie, G. H. (2023). *Egypt. J. Chem.* **66**, 167–185.

[bb12] Mohamed-Ezzat, R. A. & Elgemeie, G. H. (2024*a*). *BMC Chemistry* **18**, 58 (https://doi. org/10.1186/s13065-024-01164-9). 10.1186/s13065-024-01164-9 PMC10967038 38532431

[bb13] Mohamed-Ezzat, R. A. & Elgemeie, G. H. (2024*b*). *Nucleosides Nucleotides Nucleic Acids* **43**, 1511–1528. 10.1080/15257770.2024.2341406 38753464

[bb14] Mohamed-Ezzat, R. A., Elgemeie, G. H. & Jones, P. G. (2024). *Acta Cryst.* E**80**, 120–124. 10.1107/S2056989023011076 PMC10848973 38333139

[bb15] Mojzych, M., Ceruso, M., Bielawska, A., Bielawski, K., Fornal, E. & Supuran, C. T. (2015). *Bioorg. Med. Chem.* **23**, 3674–3680. 10.1016/j.bmc.2015.04.011 25921266

[bb16] Rigaku OD (2024). *CrysAlis PRO*. Rigaku Oxford Diffraction, Yarnton, England.

[bb17] Sheldrick, G. M. (2015*a*). *Acta Cryst.* C**71**, 3–8.

[bb18] Sheldrick, G. M. (2015*b*). *Acta Cryst.* A**71**, 3–8.

[bb19] Smirnov, V. V., Kiprianova, E. A., Garagulya, A. D., Esipov, S. E. & Dovjenko, S. A. (1997). *FEMS Microbiol. Lett.* **153**, 357–361. 10.1111/j.1574-6968.1997.tb12596.x 9271863

[bb20] Supuran, C. T. (2008). *Nat. Rev. Drug Discov.* **7**, 168–181. 10.1038/nrd2467 18167490

[bb21] Westrip, S. P. (2010). *J. Appl. Cryst.* **43**, 920–925.

[bb22] Wykoff, C. C., Beasley, J. N., Watson, H. P., Turner, K. J., Pastorek, J., Sibtain, A., Wilson, D. G., Turley, H., Talks, K. L., Maxwell, H. P., Pugh, W. C., Ratcliffe, J. P. & Harris, L. A. (2000). *Cancer Res.* **60**, 7075–7083. 11156414

